# Positioning work related stress – GPs’ reasoning about using the WSQ combined with feedback at consultation

**DOI:** 10.1186/s12875-020-01258-y

**Published:** 2020-09-11

**Authors:** Anna-Maria Hultén, Synneve Dahlin-Ivanoff, Kristina Holmgren

**Affiliations:** Unit of Occupational Therapy, Department of Health and Rehabilitation, Institute of Neuroscience and Physiology, Sahlgrenska Academy, University of Gothenburg, Gothenburg, Sweden

**Keywords:** Focus group study, Implementation, Early intervention, Primary care, Working age, Occupational stress

## Abstract

**Background:**

General practitioners (GPs) regularly handle cases related to stress and work capacity, but often find this work difficult. However, using an assessment tool in a structured way can increase GPs’ awareness of the risk for sick leave and need of referrals to preventive measures. Today there is no established methodical practice for this in primary health care. The aim of this study was to explore GPs’ reasoning about using the Work Stress Questionnaire combined with feedback at consultation as an early intervention to reduce sick leave.

**Methods:**

A focus group study was performed with 23 GPs at six primary health care centres. The discussions were analysed based on a method by Krueger.

**Results:**

Three themes emerged. *Positioning work-related stress* describes the need to make fundamental standpoints on stress and how it should be handled, to make sense of their work concerning work-related stress. *Making use of resources* focuses on GPs performing to the best of their ability using assigned resources to treat patients with stress-related ill health, even if the resources were perceived as insufficient. *Practising daily work* focuses on the GPs’ regular and preferred way of working set against the degree of intrusion and benefits. The two related themes making use of resources and practising daily work were mirrored through the third theme, positioning work-related stress, to form an understanding of how GPs should work with patients perceiving work-related stress.

**Conclusions:**

The GPs own competence and tools, those of other professionals and the time allocated were seen as important when treating patients perceiving ill health due to work-related stress. When resources were insufficient though, the GPs questioned their responsibility for these patients. The results also indicate that the GPs viewed their ordinary consultative way of working as sufficient to identify these patients. The intervention was therefore not seen as useful for early treatment of patients at risk of sick leave due to work-related stress. However, prevention is an important part of the PHC’s responsibility, and strategies concerning stress-related ill health therefore need to be more thoroughly formulated and incorporated.

**Trial registration:**

ClinicalTrials.gov, NCT02480855. Registered 20 May 2015.

## Background

Demographic, structural and technological changes are affecting the working life and working environment. Today, work-related stress has become a vital health aspect for both the individual and society. Lost working days and premature exit from the labour market lead to significant human and economic costs [1]. In Sweden, excessive workload is the most common cause of work-related illness among both women and men [2]. The importance of finding efficient measures cannot be underestimated, especially so in times of economic crisis and precarious employments [3, 4]. Early detection and treatment of work-related stress (WRS) as well as identification of underlying factors are seen as important to avoid absence from work [5–8]. Primary health care (PHC) has an important role in this respect, as it often is the first medical contact for patients with physical or mental health complaints.

General practitioners (GPs) at primary health care centres (PHCCs) often handle cases related to stress, work capacity and sickness certification in their everyday practice [9–11]. Accordingly, they seem to have a conscious approach to negotiations of sickness certification [12]. An aggravating factor, though, is that GPs often find cases concerning work ability and sick listing difficult [13, 14]. They also report poor knowledge of the workplace environment and the patient’s actual workplace situation [14–16]. However, using a functional assessment in a structured way has been shown to increase GPs’ knowledge of their patients’ workplaces and perceived stressors [17]. In addition, raising the GPs’ awareness of the risk for future sick leave due to work-related circumstances could lead them to providing advice and referring the patient to adequate preventive measures [18]. Early screening for interacting individual and work factors could make it possible not only to identify those at risk for sick leave but also to focus on the patients’ specific problems, which can be helpful for finding suitable treatments [19].

There are several instruments used to screen for stress in the workplace. Today, though, there are few instruments targeting the risk for sick leave, and there is no established methodical practice for this in PHC settings. One tool that could be of interest to use in PHC is the Work Stress Questionnaire (WSQ). It was developed in a PHC context and designed to identify persons at risk for sick leave due to WRS [20]. In addition, it has a transactional perspective, as it takes the interdependence between personal and environmental characteristics into account. The self-assessment questionnaire consists of 21 questions concerning four areas: indistinct organization and conflicts, individual demands and commitment, influence at work and work interference with leisure time [20]. In previous studies, the WSQ was found to identify WRS and to predict sick leave [21–23]. Thus, for people who consult PHC because of common mental disorders and subjective physical health complaints, the WSQ could serve to identify those at risk for sick leave. Combined with feedback at consultation, the WSQ might therefore become a preventive intervention in PHC.

Prevention is an important part of the PHC’s responsibility [24, 25]. Considering the high level of WRS, prevention of consequent ill health will become an even larger part of the PHC’s mission. Hence, the PHCCs need adequate interventions and ways to collaborate. Understanding the prerequisites for implementing an intervention is therefore of major importance for turning it into practice. Hence, the aim of this study was to explore GPs’ reasoning about using the WSQ combined with feedback at consultation as an early intervention to reduce sick leave.

## Methods

A focus group study was designed to investigate GPs’ reasoning about the systematic use of the WSQ, combined with feedback at consultation as a brief intervention. This qualitative methodology involves group discussions and is distinguished from other qualitative group interviews by the explicit use of group interaction to collect data on a specific research topic [26]. Epistemologically, the methodology shares some basic assumptions with social constructivism [27], as the focus group members jointly construct a frame of reference by which to understand their experiences, thereby leading to the development of new knowledge in interaction, seen as a learning process. By sharing experiences with one another, discussing different standpoints and asking each other questions, the participants help the researcher to achieve new knowledge [26].

### The design and setting of the brief intervention

The brief intervention was tested in a two-armed randomized control trial (RCT), to evaluate whether systematic use of the intervention could serve as a method for GPs to prevent or reduce patients’ sick leave due to WRS. The RCT was conducted at seven PHCCs located in both urban and rural areas in the region Västra Götaland in Sweden. The WSQ was used with permission from the creator. The trial has been previously described in detail in a study protocol [28] and in research articles [29–31]. The intervention included four parts: (1) participating GPs received a brief training session to gain knowledge about handling the results from the WSQ and to support their awareness about WRS; (2) the patient filled in the WSQ before consultation with the GP to identify WRS and to aid to the patient’s self-reflection; (3) the patient received feedback on the WSQ results from the GP to motivate the patient to address the work situation if warranted; and (4) the GP and the patient discussed and initiated preventive measures, if needed [32]. The intervention was carried out during either a drop-in appointment or a 30- to 45-min scheduled appointment. The number of interventions carried out by each PHCC varied between 8 and 41, with an average of 21.

### Study participants

The focus group discussion targeted the GPs participating in the intervention group included in the RCT [28]. GPs at six out of seven PHCCs were included. The last centre was excluded from the study due to a low number of patients taking part in the RCT. Four out of six PHCCs were publicly run. Twenty-six intervention GPs were invited to participate, of whom 23 accepted, 12 men and 11 women. Three GPs had to decline due to parental leave or leaving their positions. Fifteen of the participants included were general practitioners, and eight were resident physicians. Prior to the intervention period, the research team visited the participating PHCCs and presented the RCT, including the focus group study. During the intervention period, the research team also held brief training sessions with the GPs as part of the intervention. The participants received written information about the study and provided their written informed consent for the focus group study. Participants were also informed of their right to withdraw from the study at any time and were given assurances about the confidentiality of their contribution. Ethical approval was obtained for the study from the Regional Ethical Review Board at the University of Gothenburg, Sweden (reference number 125–15).

### Focus group procedure

After the intervention period was completed at a PHCC, the intervention GPs at that particular PHCC took part in a focus group discussion to explore their reasoning about the systematic use of the WSQ. The discussions followed a semi-structured discussion guide within four prioritized areas: the content of the intervention, preparations and peripheral resources, the use of the intervention in daily work and the prerequisites for future implementation and use in the PHCC. A copy of the discussion guide is presented in Additional file 1. As communication between the participants is decisive for the outcome and the group process, the moderator encouraged the participants to clarify their reflections and reasoning. The group discussions were held at the PHCCs and were moderated and co-moderated by the second (SDI) and third authors (KH), both of whom are experienced in focus group methodology. The role of the moderator was to create an open and friendly atmosphere in which the participants could feel free to express their views [33] and to encourage the participants to share their experiences [34]. The group sessions were audiotaped and transcribed verbatim. As shown in Table 1, each focus group included two to five participants, and the discussions lasted between 28 and 44 min. The discussions were held between 23 October 2015 and 10 March 2016.

**Table 1:** Focus group characteristics and length of discussion

Focus group	Primary health care centre provision	Number of participants	Number of participating women/men	Length of discussion (minutes)
1	Public	3	1/2	44
2	Public	5	1/4	29
3	Private	2	1/1	28
4	Public	4	3/1	38
5	Public	4	3/1	36
6	Private	5	2/3	40

### Analysis

The analysis was based on the focus group method formulated by Krueger [32]. It was explorative, driven by the problem in hand and held close to the raw data. Initially, the researchers listened to the audiotapes and read the transcripts repeatedly, to become more acquainted with the entire raw data. Individual discussions relating to the problem were then broadly grouped, with recurrent perspectives forming the base for preliminary themes. The texts within each preliminary theme were then read through and sorted into categories, representing different aspects of the theme. The parts, both the themes and the categories, were seen in relation to the whole, making it necessary to revise and reformulate them in parallel to reading transcribed texts and listening to recordings. Thereafter, the categories were summarized and abstracted, to get a descriptive overview as a preparation for the final step, the interpretation, which was an overarching process that began during the discussions, to capture the essence of the discussions. The first author performed the analysis in close conjunction with the third author. Throughout the entire analysis process, the three authors discussed the meaning and interpretation of the data in order to reach agreement, thereby exploring and verifying their different understandings of the discussions. The software NVivo 12 was used to organize, store and retrieve data, and additionally to categorize while still staying close to the raw data, and to enable backtracking.

## Results

**Fig. 1:**
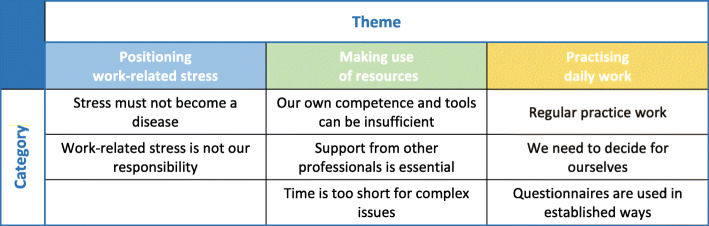
The three themes and eight categories describing the reasoning about using the intervention

The analysis showed that the intervention was seen in relation to three major areas, labelled as themes: positioning work-related stress, making use of resources, and practising daily work. Positioning work-related stress describes the need to make fundamental standpoints on how stress should be handled. Making use of resources focuses on GPs performing to the best of their ability with assigned resources. Practising daily work describes the GPs’ regular and preferred way of working. The themes, along with their eight associated categories, are presented in Fig. 1 and described below.

**Fig. 2:**
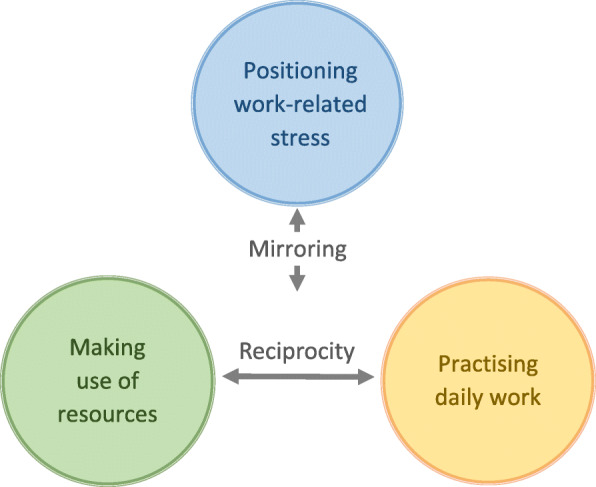
Interrelating aspects affecting the brief intervention in the context of the primary health care

The three themes form the basis for reasoning about whether and how to integrate and use the intervention in practice. The reciprocal relationship between the themes making use of resources and practising daily work was fundamental for the participants’ opinions about whether and how the intervention could be considered useful during consultations. The two related themes were mirrored through the third theme, positioning work-related stress, to form an understanding of how to treat patients perceiving WRS (Fig. 2).

### Positioning work-related stress

The intervention highlighted the importance of how GPs should work with issues concerning WRS, and whether or not they should screen for ill health as a consequence. Therefore, it was necessary to position stress in general as well as the GP’s role and responsibility to prevent and treat stress-related ill health.

#### Stress must not become a disease

Understanding how stress originates was fundamental for deciding when stress becomes a medical issue and how to apply the intervention. Stress was considered to be prevalent in society, and a general problem and phenomenon in the spirit of the age. The participants viewed work as a plausible cause of stress, but stress in general was seen as a lifestyle problem stemming from a working situation and a private life that were incompatible.

*P2 It’s not just a case of work-related stress, but lifestyle stress. So even if they are on sick leave, there’s still talk about working full time and then there’s three children and a partner …*

*P1 And then work out, meet my friends and have dinner [laughter]*

*P2 Right, and yoga, of course...*

*P1 It feels like a lifestyle situation …*

*P4 … that’s being made into a sickness.*

*P1 Quite right, absolutely.*

*Group 4*

The participants concluded that stress is not a disease or a medical issue, but its negative effect on health is. The participants emphasized the importance of not turning stress into a disease due to its commonness. However, by screening for WRS, general practitioners might bring it into focus and thereby increase the risk of turning stress into a medical issue.

#### Work-related stress is not our responsibility

The GP’s part in the early identification and treatment of WRS was questioned. The participants considered GPs to have a role based on their competence and experience to treat the patients in question. However, the responsibility should be limited to treating the medical health problems caused by stress. Hence, the participants saw a possible dilemma, as problems in the workplace might remain unsolved and actions taken by the GP become ineffectual in a longer perspective. As WRS was seen as a lifestyle problem, a large responsibility was put on the individual to handle stress-related symptoms. Still, they considered that help must be within reach. Therefore, when the cause of ill health due to stress is found at the workplace, entirely or in part, the employer and the occupational healthcare service, but also the trade union and the National Insurance Office, must take part and assume a larger responsibility than happens today. In addition, the participants were not totally in favour of GPs screening for patients experiencing WRS, as this might increase the GPs’ responsibility even more.

### Making use of resources

Ill health due to WRS was seen as a complex issue requiring specific and coordinated resources. However, the participants’ capacity to make use of assigned resources, divided into their own competence and tools, those of other professionals and the time allocated, could be perceived as challenging. Hence, the participants had to work to the best of their own abilities with these resources, thereby questioning if there are sufficient resources throughout the PHC to handle these issues and use the results from the WSQ effectively.

#### Our own competence and tools can be insufficient

The participants’ medical competence, tools and areas of interest formed a foundation for their work, but also constituted a delimitation of their actions, hence, affecting their possibility to handle the results of the WSQ. When they considered themselves to have sufficient experience and suitable tools to treat patients with stress-related ill health, the results of the WSQ were useful. For instance, the results were used to gain an understanding of the problem, but also when discussing, assessing the need for, and proposing suitable measures. However, when the participants felt they did not have the necessary competence and applicable tools, it was difficult to interpret and act on the results from the WSQ.

*P3 I wonder what was expected of me … I’ve done what I think I’ve been able to do. Then, I’ve no idea about how others have acted.*

*P1 I ask for the next step … I was not sure, OK, you have to analyse and see the results. And then, what’s the next step?*

*P3 I believe it’s quite normal, as when I get a new task and it’s something new, I’d like to have it structured in a certain way; this is expected ... It worked out in any case, so there’s nothing untoward about that, but I’d like to have had a bit more clarity.*

*P2 I’d understood it as something that would help me to put focus on these matters. Then, of course, it would have made things easier if we’d have had more knowledge about how to proceed, as you’d said – how do we proceed ...?*

*Group 1*

In addition, they had doubts about the effect of their accepted measures, that is, education, sick listing, counselling and supportive conversations.

#### Support from other professionals is essential

The accessibility to other health care professions linked to the PHCC was important. Having low accessibility meant not being able to suggest appropriate measures based on the results from the WSQ. It also meant not being able to help patients with stress-related ill health in the longer-term, in turn, reducing the participants’ trust in other professions and inducing a sense of hopelessness. Thereby, the willingness to handle cases concerning WRS decreased. Instead, having a well-established cooperation increased the possibility of handling the results from the WSQ. Seen in a larger perspective, it induced a sense of being part of a team with joint resources concerning stress-related ill health. The cooperation emanated from an understanding of and belief in the competence of these professions to handle such issues. This instilled the participants’ hope of being able to help patients in the long turn, increased their willingness to handle cases concerning WRS and strengthened them as professionals.

*P2 It’s pretty good, at least, at the moment I think.*

*P1 Yes, [we’ve got] a psychologist, an intern psychologist and a counsellor, where the psychologists together work 170%–180% or thereabouts.*

*P2 Yes, and we’ve got an occupational therapist, too …*

*P1 Then we have a social worker employed at 50%, who combines both support in the sick leave process and conversational support and a rehab coordinator role, so we have good help there, too.*

*P2 That’s a really good thing.*

*P1 So, as doctors, we’re not alone in this.*

*Group 3*

#### Time is too short for complex issues

The participants perceived a lack of time to address stress-related issues in detail or to raise questions about a patient’s working life in a way that the intervention required. In general, working within narrow time frames was considered as a limiting factor for practising the profession. They felt they had insufficient time to carry out what was expected. In addition, the participants constantly had to consider what needed to be investigated during the session and how the necessary information could be acquired as quickly and efficiently as possible. Therefore, time was considered too short to handle cases concerning such complex issues as stress-related ill health. Redistributing time was not seen as a solution, as this only affected other groups of patients negatively. Hence, allocating time for the intervention in hand was therefore not seen as realistic under prevailing circumstances.

### Practising daily work

The participants had developed their own work procedures for their consultations, emanating from the cause for seeking help, the patient’s narrative and the GP’s responsibility to exclude serious illness. Performing the intervention could alter these procedures and therefore be perceived as either positive or negative or both, depending on the degree of intrusion into daily work set against the benefits. The intervention was seen in relation to the GPs’ regular procedures for diagnosis and the need to make decisions based on their judgements, as well as their established way of working with questionnaires.

#### Regular practice work

In performing the intervention, the participants’ attention was directed to a greater extent towards the prevalence of WRS, but also towards perceived contributing factors. However, they considered themselves to have a well-functioning work procedure for diagnosis and considered it applicable for patients with illness due to WRS as well. They argued that there is a general awareness of stress in society and that that there is no stigma attached to it. Hence, they expected to be informed by the patient if the perceived problems were stress-related and if work was a probable cause. By listening to the patient’s narrative and asking relevant questions, the participants were made aware of any stress-related ill health. Therefore, they rarely needed to ask specifically about stress-related ill health or the work situation. At the same time, the general use of questionnaires was questioned. Talking to the patient was perceived to give the information needed, making questionnaires redundant.

*P2* There’s a lot of stress-related ill health that we face, and I feel that I’d get that information anyway. It [the intervention] would only complicate the consultation, so I don’t think I’d use it in that way, no. If I suspect there’s an issue, we’ll take that face to face ...

P5 We’ve been trained from the outset to put open questions, getting the patients to tell their own story. These questionnaires make this process somewhat more unnatural ...

P3 I think many of the questions in the questionnaire are asked, anyway. If it is stress-related, you ask what the problem is and try to delve deeper. I think, as you say, that the information will be forthcoming anyway.

*Group 2.*

#### We need to decide for ourselves

The participants felt they wanted to decide for themselves when and how to use the WSQ. Starting the consultation by letting the patient fill in the self-assessment questionnaire could affect the progress of the consultation negatively. For instance, it could steer the participants too speedily towards WRS and risk missing serious illness.

*P2 If the patient gets high WSQ scores, at once you might unconsciously think it’s work-related stress.*

*P4 On the contrary, it might otherwise be the case that you might have missed it more if you hadn’t had that knowledge.*

*P2 Even so. A patient that I’m thinking of sought care for chest pains and had high WSQ scores. Then you swiftly proceed in that direction, and it turned out to be right. But afterwards, I thought, I may have unconsciously been less thorough in the cardiac anamnesis and rushed things along.*

*Group 6.*

Depending on the patient’s perceived problems, the participants could find it hard to relate to and use the results from the WSQ naturally in the conversation. Hence, the participants viewed the WSQ as a potential extra tool to analyse WRS, as they saw positive effects of using it, such as structuring the conversation, giving an overall view of the problem as well as harmonizing work and improving its quality. Altogether, the participants emphasized that the GPs must be able to judge when to use it, based on the prevailing situation and their preferred way of working.

#### Questionnaires are used in established ways

As the participants already had their own established ways to use questionnaires, they thought that the WSQ should be added to those procedures, rather than including it in a fixed intervention. For instance, questionnaires were used as screening tools for selected groups or when the participant had an idea of the area of concern. If the questionnaires were quick to use or the matter was considered delicate, they could be given to the patient to be filled out during a consultation. Otherwise, they were filled out in the waiting room after the consultation or taken home to be filled out for the next consultation. Also, different questionnaires could be used in combination, if deemed relevant. In addition, the participants weighed different questionnaires against each other, to decide which to use and when to use them. There were different incentives for selecting tools, for instance, a clear and relevant result, experience of using the questionnaire, financial compensation, amount of time and ease of interpretation.

## Discussion

The brief intervention was seen in relation to the participating GPs’ understanding of how to help people with ill health due to WRS on an overall level (*positioning work-related stress*). In addition, they questioned whether the PHC’s resources altogether were sufficient to prevent and treat ill health due to WRS (*making use of resources*) and were critical of the effect that the intervention could have on their well-functioning way of working (*practising daily work*). Therefore, performing the intervention meant relating to issues even beyond the PHC setting. The discussions were therefore not primarily centred on the intervention or the WSQ, as such. Instead, they focused on taking concurrent factors on the societal, organizational and individual levels into consideration when implementing the tested or a similar intervention.

The framing of work-related stress and the importance of not turning stress alone into a disease was emphasized in this study. Stress was viewed as an individual lifestyle problem rather than a sign of potential ill health, even if circumstances not directly related to the personal level could affect the work-related ill health. For instance, work-changes induced by economic crisis were found to affect work-related common mental distress [3] and precarious employments were found to increase the risk of receiving a disability pension [4]. One reason for emphasizing the importance of not turning stress into a disease could be the risk of medicalizing everyday stressful events, as this might lead to overdiagnosis and additional strains for the health care sector. Therefore, when GPs identify and treat negative aspects of WRS, they have to consider whether the stressful events perceived by the patient are to be seen as normal difficulties in everyday life. As a measure to prevent medicalization, Parker et al. [33] recommend that messages that promote medicalization of normal mental states and imply individual responsibility for mental well-being should be challenged in the clinician–patient relationship. However, the framing of these conditions to a large extent takes place outside this relationship [34], making it necessary to take the dynamics between macro and micro levels of medicalization and the influence of multilevel incentives into account [35]. Consequently, a position needs to be adopted about which factors and overarching strategies are seen as crucial in preventing stress-related ill health and in treating individuals with the condition [5, 8, 36]. The results from this study indicate that such factors and strategies need to be more thoroughly formulated and incorporated.

Having indistinct, overarching strategies about the handling of stress-related ill health could lead to uncertainty about the division of responsibilities concerning WRS. In accordance, prevention and early treatment of WRS was not seen in this study as an obvious responsibility of GPs, even if prevention is declared to be an important part of the PHC mission [24, 25]. A possible explanation is that preventive health care in Sweden is focused on behavioural risk factors concerning smoking, unhealthy diet, risky alcohol consumption and physical inactivity, while other important factors, such as WRS [37], are less highly prioritized. Further, the participants considered that the actors liable for the work environment should have a larger responsibility for handling the consequences of WRS. A reason for this view could be that occupational health care is thought to be better suited and more qualified than PHC to deal with it [38], despite the fact that services provided through occupational health care in Sweden can be very limited. Therefore, it might be necessary to clarify the division of responsibilities between different actors.

Based on the study findings, the participant’s own competence and tools could be perceived as insufficient when performing the intervention. This is in line with earlier studies showing that GPs lack competence to use assessment tools [39] and find it hard to fulfil their competence roles in cases concerning work ability and sickness certification [13, 14]. This means that the GP’s degree of versatility could be taxed to its limit. Further, the amount of training on a topic and the self-confidence about a topic have been found to affect the prevention-related clinical practices [40]. Not having sufficient competence and finding it hard to fulfil their roles could make it hard to identify and treat patients with WRS. Considering the above-described mirroring process, lacking competence could also be a general dilemma, as it could affect GPs’ overarching view of the responsibility for handling ill health due to WRS.

According to this study, the participants’ own competence as well as time and support from other professions were essential when handling such a complex issue as stress. Having these resources increased the participants’ willingness to handle cases concerning WRS and strengthened them as professionals. The GPs’ own stressful work situation is, however, a problem [41, 42], not just because the work situation affects them personally, but also because it increases the likelihood of making errors and delivering suboptimal patient care [43]. Feeling overworked can also lead to a reduced ability to offer empathy, compassion and support to patients [44, 45]. In addition, GPs having prevention-related healthy habits themselves has been found to affect their self-reported prevention-related counselling and screening practices [40]. For the GPs to be able to handle and engage in different aspects of the patient’s ill health due to WRS, their own work situation must be manageable.

Using the brief intervention meant reflecting on the degree of intrusion into their perceived properly functioning way of working. This is in line with a Swedish study [39], where GPs were found to perceive the use of a depression self-assessment scale as more of a hindrance than a help to diagnosis. The participants also reflected on the risk of making the wrong diagnosis when using the intervention. According to a British study [46], GPs used a set of stages and strategies to make diagnoses, but the way these were used differed, as the strategies were transformed according to each GP’s personal style [47]. Hence, not only the diagnostic procedure but also the motives for diagnostic decision-making [48] can explain the variation in the degree of intrusion perceived in the study at hand. Further, findings from a Dutch study [49] indicate that merely training GPs about work-related problems does not to improve GPs’ registration of work-related problems and occupation. It might therefore be of value to take greater account of the GPs’ own developed ways of working and motives for diagnostic decision-making, when implementing an intervention.

The results show that the participants preferred the patient’s narrative as a basis for the consultation and therefore found the intervention redundant. According to Davidsen and Reventlov [50], a narrative approach could lead to enhancement of the GP’s empathy and a broader understanding of patients suffering from psychological problems, and to improvement of the patients’ recovery by enhancing their own agency. However, patients might hesitate to reveal that they are unable to cope with stress [51]. Using narratives also implies a challenge for the GPs, as they face the complex task of motivating their patients to achieve optimal health while also ensuring their satisfaction [52]. In addition, as people with WRS sometimes are thought to be in denial or unaware of the stress-related problem underlying their perceived symptoms, the GPs might see problems other than those described by the patient. The understanding of the message communicated by the patient is also relevant to consider. Findings from a study on a general population showed that an individual’s own stress mindset affected the judgement of others’ work strains, that is, the likeliness of judging an individual experiencing a heavy workload as suffering from burnout, somatic symptoms or presenteeism [53]. The GP’s mindset could therefore have consequences for the possibility of identifying work-related stress early and taking necessary measures.

In summary, the study revealed that there were important considerations about the intervention in relation to views about WRS, PHC’s responsibilities and resources as well as GPs’ preferred ways of working. However, it is also necessary to listen to concerned parties other than the GPs and include other contextual aspects, to understand the prerequisites needed to use a new tool. Based on a Swedish study [54], a positive creative climate indicated more frequent use and positive perceptions by GPs regarding use of a new tool than did the least positive creative climate. Implementing and using the studied intervention requires considerations of the specific PHCC context and also overarching premises, as well as the relationship between these parts. Therefore, there is a need to question what should be done to handle a phenomenon and a diagnosis that does not fit into the present system and ways of thinking about preventing and treating ill health and disease.

### Strengths and limitations

The focus group method is based on the collective understanding of the participants’ views emerging during the discussions. To achieve the best conditions for this understanding to emerge, human interaction was encouraged. The moderator made sure that all participants took part in the discussions, but also raised relevant questions and made comments when necessary. In addition, the participants in each focus group shared the experience of working as GPs and of having performed the intervention, thereby ensuring that they had common ground and shared experience as a basis for their discussion. Contradicting views were respected and considered valuable, which supported the participants to raise and discuss a wide spectrum of opinions and reflections as well as to reason and draw conclusions about different topics. Further, the moderators were experienced and comfortable in the role as group leaders.

Even though efforts were taken, there were limitations that have to be considered. The study was conducted in one county council. There is therefore a risk that the PHCCs in other county councils have other prerequisites for their work, thereby limiting the transferability of the results. In addition, there has to be a degree of heterogeneity to ensure a vivid discussion. Normally, the composition of the groups is carefully arranged to obtain a broad representation of the target group and an open atmosphere that stimulates group discussions. As the study was set to evaluate the reasoning about using the intervention tested in the RCT, the sample was limited to those GPs. Since they worked at the same PHCC, the possibility to consider heterogeneity was limited. Even so, the groups were heterogeneous when taking gender and professional experience into account. Increased heterogeneity could have been achieved by putting together mixed groups, with GPs representing different PHCCs. However, that was not possible in practice. The fixed set of participants made it less relevant to consider an appropriate sample size. However, the number of focus groups was found to be sufficient, as no new topics were raised in the last two discussions. Further, the number of participants in each group discussion was small. However, focus groups consisting of up to six participants have been found to be preferable to larger groups [32], as they give greater opportunity for dynamic discussions and allow all the participants to express their views. The group discussions took place soon after the intervention was completed. Even so, for a few participants it was hard to recall their performance of the intervention, especially if they had conducted few interventions.

To ensure the quality of the focus group analysis, a method developed by Krueger was used, as it is well established. In addition, there was a risk of confirmation bias, since the third author had developed the WSQ. As all three authors were included in the analysis process to confirm and contest each other, the risk of confirming beliefs and producing results based on preconceptions was decreased. In addition, fellow researchers as well as professionals working in the field of PHC have scrutinized the preliminary results and brought the analysis forward.

## Conclusions

The results from this study indicate that concurrent factors on the societal, organizational and individual levels have to be taken into consideration when implementing the tested or a similar intervention. The framing of stress and the understanding of PHC’s responsibility for patients perceiving ill health due to WRS were seen as fundamental. Further, the GPs own competence, the support from other professions and the time allocated were considered important when handling such a complex issue as stress. The responsibility to handle patients that perceive ill health due to WRS was even questioned when the resources were insufficient. The results also indicate that GPs perceived no need for tools to identify these patients, as they view their regular consultative way of working as sufficient. The intervention tested was therefore not considered useful or necessary for early treatment of patients at risk of sick leave due to WRS. However, PHC has a central role in the health care system and within this a responsibility for prevention. Accordingly, strategies to prevent and treat individuals with stress-related ill health need to be more thoroughly formulated and incorporated.

## Supplementary information

**Additional file 1.** Discussion guide, Microsoft Word Document DOC, A semi-structured discussion guide used during the focus group discussions.

## Data Availability

The datasets used and analysed during the current study are available from the corresponding author on reasonable request.

## Abbreviations

WSQ: Work Stress Questionnaire
GP: General practitioner
PHC: Primary health care
PHCC: Primary health care centre
WRS: Work-related stress
