# Supplementary material for: Positioning work related stress – GPs’ reasoning about using the WSQ combined with feedback at consultation
Source: BMC Fam Pract. 2020 Sep 11;21:187. doi: 10.1186/s12875-020-01258-y (PMC7488670; doi:10.1186/s12875-020-01258-y)
Supplement: Supplementary file 1 — Additional file 1. Discussion guide, Microsoft Word Document DOC, A semi-structured discussion guide used during the focus group discussions. [file 12875_2020_1258_MOESM1_ESM.docx]

**Discussion guide**

**A semi-structured discussion guide used during the focus group discussions**

**The content of the intervention**
What do you think about the content of the intervention?

1. Patients filling in the WSQ
2. Feedback on results
3. Discussions and initiation of preventive measures

**The use of the intervention in daily work**

1. What are your views of the WSQ to be used as a measure for identification of patients at risk of ill health due to work-related stress?
2. What are your views of the WSQ to be used as a tool for general practitioners in primary health care?

**The prerequisites for future implementation and use in the PHCC**

1. What are the possibilities for using the WSQ in daily primary health care practice?
2. What are the barriers for using the WSQ in daily primary health care practice?

**Preparations and peripheral resources**
What do you think about the administration and conduct of the study?

1. Information about the intervention, the WSQ and the intervention received before the conduct of the RCT
2. The work performed by the research assistant
3. Receiving a summary of the results on a paper in conjunction to the consultation
